# Evidence of cryptic lineages within a small South American crocodilian: the Schneider’s dwarf caiman *Paleosuchus trigonatus* (Alligatoridae: Caimaninae)

**DOI:** 10.7717/peerj.6580

**Published:** 2019-03-22

**Authors:** Pedro Senna Bittencourt, Zilca Campos, Fábio de Lima Muniz, Boris Marioni, Bruno Campos Souza, Ronis Da Silveira, Benoit de Thoisy, Tomas Hrbek, Izeni Pires Farias

**Affiliations:** 1 Laboratory of Animal Genetics and Evolution (LEGAL), Federal University of Amazonas, Manaus, Amazonas, Brazil; 2 Graduate Program in Genetics, Conservation, and Evolutionary Biology, National Institute of Amazonian Research (INPA), Manaus, Amazonas, Brazil; 3 Wildlife Laboratory, Brazilian Agricultural Research Corporation (EMBRAPA) Pantanal, Corumbá, Mato Grosso do Sul, Brazil; 4 Graduate Program in Freshwater Biology and Inland Fisheries, National Institute of Amazonian Research (INPA), Manaus, Amazonas, Brazil; 5 Chico Mendes Institute for Biodiversity Conservation (ICMBio), Boa Vista, Roraima, Brazil; 6 Laboratory of Zoology Applied to Conservation, Federal University of Amazonas (UFAM), Manaus, Amazonas, Brazil; 7 Institut Pasteur de la Guyane, Cayenne, French Guiana; 8 Association Kwata, Cayenne, French Guiana

**Keywords:** Jacaré-coroa, Conservation genetics, Amazonian crocodilians, Biogeography, Population genetics, Diversification

## Abstract

Schneider’s dwarf caiman *Paleosuchus trigonatus* is one of the smallest living crocodilians. Due to its broad distribution, cryptic behavior, and small home range, the species is well suited for the study of phylogeographic patterns on a continental scale. Additionally, this species is under threat due to habitat loss, trade and harvest, but is considered at low conservation risk by the IUCN. In the present study we test the hypothesis that *P. trigonatus* is comprised of geographically structured lineages. Phylogenetic reconstructions of the mitochondrial cytochrome b gene and single locus species discovery methods revealed the existence of two well-supported lineages within *P. trigonatus*—an Amazonian and Guianan lineage. Fossil calibrated divergence of these lineages was estimated to have occurred in the Late Miocene (7.5 Ma). The hypothesis that the Atlantic coast drainages might have been colonized from the southeast or central Amazon is supported by demographic metrics and relatively low genetic diversity of the Coastal and upper Branco populations when compared to the Amazon basin populations. The Amazon basin lineage is structured along an east-west gradient, with a sharp transition in haplotype frequencies to the east and west of the Negro and Madeira rivers. These lineages are already under anthropogenic threat and, therefore, are conservation dependent. Recognition of these lineages will foster discussion of conservation future of *P. trigonatus* and these lineages.

## Introduction

The importance of knowing the spatial distribution of genetic diversity of “widely” distributed species cannot be underestimated. Current spatial organization of diversity is a window to the past and, through phylogenetic analysis, provides insights into the history of taxa (Futuyma & Agrawal, 2009). Moreover, this knowledge allows the detection of distinct evolutionary lineages or even cryptic species and ultimately guides management and conservation efforts of these taxa.

Distinct evolutionary lineages and cryptic species are frequently discovered without *a priori* intention during phylogenetic, phylogeographic, and population genetic surveys (Pfenninger & Schwenk, 2007), and are not restricted to poorly known or small and obscure taxa. They are also observed in large charismatic taxa (e.g., river dolphins (Hrbek et al., 2014), giant Galapagos tortoises (Poulakakis et al., 2015), tegu lizards (Murphy et al., 2016), and silky anteaters (Miranda et al., 2017)). These cases are particularly common in the tropics whether in the Neotropics (see above) or in the Paleotropics (e.g., African elephants (Roca et al., 2001), species of giraffes (Fennessy et al., 2016), a new species of orangutang (Nater et al., 2017)).

African crocodiles of the genus *Osteolaemus* also comprise three deeply divergent molecular lineages (Eaton et al., 2009) subsequently delimited but not described as new species by Shirley et al. (2014). These *Osteolaemus* lineages are morphologically divergent (Brochu, 2007; Eaton et al., 2009). *Crocodylus niloticus* and the resurrected *C. suchus* were unambiguously discriminated using DNA data and, although these data are not yet published, also show fixed discrete and non-overlapping continuous character variation (Hekkala et al., 2011). The genus *Mecistops* also contains two lineages delimited as new species by Shirley et al. (2014), and a recent revision of the genus resulted in the revalidation of *Mecistops leptorhynchus* for one of the lineages, with the other lineage being *Mecistops cataphractus* (Shirley et al., 2018). More recently, South American crocodilians were also shown to possess intraspecific lineages with highly restricted gene flow. Muniz et al. (2018) have shown that the Cuvier’s dwarf caiman *P. palpebrosus* comprises at least three lineages—Evolutionarily Significant Units (ESUs) occupying the Amazon, Madeira-Bolivia and Pantanal basins—and the authors speculate that *P. palpebrous* could be a complex of cryptic species hidden under the same scientific epithet. Independent of being recognized as distinct species or formally described as such, these crocodilian lineages are the direct outcome of evolution, and thus their recognition and study is necessary to comprehend the evolutionary history of this group. Only by focusing on lineages rather than on described species, can we hope to understand the evolutionary history of the studied group (Willis, 2017).

The Schneider’s dwarf caiman *Paleosuchus trigonatus* (Schneider, 1801), the sister species of *P. palpebrosus*, is one of the smallest of living crocodilians (Magnusson, 1989). It inhabits small streams and rivers within densely forested habitats (Magnusson, 1992), open waters often near rapids (Vasconcelos & Campos, 2007), igapó forests—seasonally flooded forests on white sands soils—of central Amazon (Souza-Mazurek, 2001), the Branco River-Rupununi savannas (Muniz et al., 2015), and the transitional forest of the Amazon/Cerrado biomes in the headwaters of the Juruena River (Campos, Mourão & Magnusson, 2017). Despite its wide distribution, *P. trigonatus* is rarely observed. In central Amazon, adults spend most of their time in terrestrial retreats, hidden in burrows, hollow logs and debris cavities under fallen trees. They occur at low densities and have a small home ranges, with males being strongly territorial (Magnusson & Lima, 1991; Campos, Muniz & Magnusson, 2017). Major threats to the species include habitat destruction, deforestation, mining, and highway/road construction, which in turn leads to urbanization, pollution and local hunting (Magnusson & Campos, 2010; Campos, Muniz & Magnusson, 2012). Hydroelectric dam construction and the filling of the dam also leads to habitat and nesting site destruction, leading to the displacement of individuals into areas where the risk of mortality is increased or areas with less nesting habitat (Campos, Mourão & Magnusson, 2017).

Although phylogenetic relationships among caimans are well established (Hrbek et al., 2008; Oaks, 2011), there are no intraspecific genetic studies of *P. trigonatus*. Due to its broad geographic distribution, cryptic behavior, and small home range, *P. trigonatus* is well suited for testing for the presence of multiple evolutionary lineages, their spatial distribution and their evolutionary history. Therefore, we used the mitochondrial cytochrome *b* gene (cyt *b*) to calculate genetic diversity parameters, infer population genetic structure, and to test if instraspecific lineages within *P. trigonatus* exist and when they diverged, through the use of phylogenetic reconstruction, single locus species discovery and molecular calibration methods. Cytochrome *b* is a versatile molecular marker most useful for phylogenetic inference at or below the family level (Meyer, 1994; Castresana, 2001).

## Materials and Methods

### Study area and sample collection

**Figure 1:**
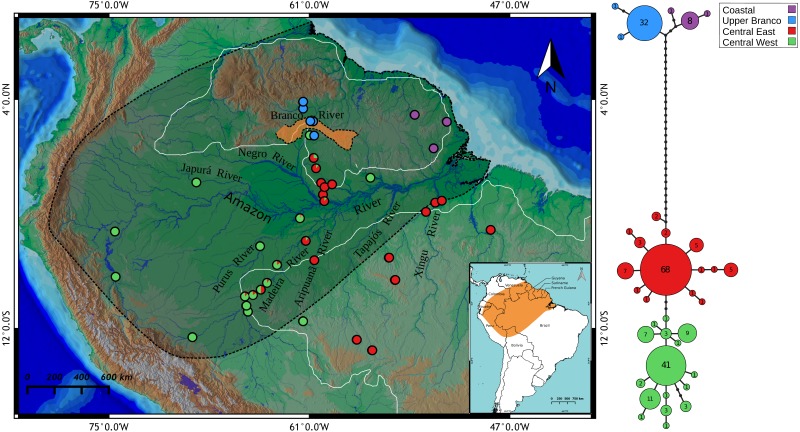
Map showing all the sites sampled in this study, results of the BAPS analysis and haplotype network based on the mitochondrial cyt b gene. The colors represent the cluster to which the analyzed individuals belong based on BAPS assignments. The points with two colors on the map indicate sites where haplotypes assigned to different groups were found. Each cluster is also highlighted in the haplotype network, indicating the Amazonia lineage (red and green) and the Guiana lineage (purple and blue). Shaded area represents the species geographic range currently accepted by IUCN (Crocodile Specialist Group, 1996). The orange highlighted area in Branco River comprises relicts of the paleodivisor Amazon/Proto-Berbice (Parima, Demini, Apiaú, Mucajaí, Mocidade, Grande, Lua, Anauá, and Acaraí mountain ranges). White boundaries to the north and south of the Amazon River represents the Guiana and Brazilian shields, respectively. The map was constructed in QGIS and the final graphic in Inkscape.

We sampled 230 *P. trigonatus* specimens from 42 localities within the known distribution of the species (Fig. 1). The northern–most locality sampled lies in the Branco River basin (Amajari –3°50′N, 61°26′W), the southern–most in the Juruena River basin (São José do Rio Claro –13°31′S, 56°37′W), western–most in the Japurá River basin (Japurá River –1°50′S, 69°20′W), and the eastern–most in the Tocantins River basin (São Pedro da Água Branca –5°60′S, 48°20′W). We also included samples from Peru (Pacaya–Samiria National Reserve –5°13′S, 74°34′W; Puerto Maldonado –12°35′S, 69°10′W; Ucayali River –EU496862, (Venegas-Anaya et al., 2008)), and French Guiana (Tampok River –2°55′N, 53°38′W). All localities, coordinates, and number of samples are listed in Table S1.

We removed one or two tail scutes of adults, subadults or juveniles after capture and marking during nocturnal surveys, stored them in tubes containing 95% ethanol and deposited them in the Universidade Federal do Amazonas animal tissue collection (CTGA) under individual ID tags. We captured and sampled all individuals under license granted by the Instituto Brasileiro do Meio Ambiente e dos Recursos Naturais Renováveis (IBAMA, permits #49641-2 and #13048-1). ICMBio/IBAMA field collection permits are conditional that collection of organisms be undertaken in accordance with the ethical recommendations of the Conselho Federal de Biologia (CFBio; Federal Council of Biologists), Resolution 301 (December 8, 2012). Access to genetic resources was authorized by permit number 034/2005/CGEN.

### Molecular data

We extracted whole genomic DNA using 2% CTAB solution (2% CTAB, 1.4 M NaCl, 20mM EDTA, 100 mM Tris HCl, 1% PVP) (Doyle & Doyle, 1990), with an addiction of 15 mg/mL Proteinase K. We PCR-amplified the mitochondrial cytochrome b gene (cyt b) using primers GluCRf.1 (5′-CAACCAAAACCTGAGGYCTGA-3′), and ProCRr.1 (5′-ATTAGAAYGTCGGCTTTGGGG-3′), following protocols described by Hrbek et al. (2008). We used Exonuclease I –Shrimp Alkaline Phosphatase (ExoSAP) to purify PCR products which we then used for fluorescent dye terminator sequencing using primers CytbCRf.1 (5′-ATGACCCACCAACTACGAAAA-3′), and CytbCRf.3 (5′-CCATACATYGGAGACACCAT-3′) (Hrbek et al., 2008), following the manufacturer’s recommended protocols for ABI BigDye Terminator (Applied BioSystems, Foster City, CA, USA). We precipitated the sequencing reaction using 100% Ethanol/125 mM EDTA solution, resuspended it in Hi-Di Formamide, and resolved it on an ABI 3500XL automatic sequencer (Applied Biosystems). We organized and verified the nucleotide sequences in the program Geneious 6 (Kearse et al., 2012), using Clustal W (Thompson, Higgins & Gibson, 1996) to perform an automatic alignment followed by final verification by eye. We observed no indels. We translated sequences into putative amino acids, and no internal stop codons were found. We deposited the sequences in the GenBank under accession numbers MH757465–MH757694.

#### Single locus species discovery

For single locus species discovery analyses we first merged the alignment of *Paleosuchus trigonatus* sequences with the Cytochrome *b* alignment of *Paleosuchus palpebrosus* (MH846341–MH846546) and then reduced the dataset to unique haplotypes using the function hapCollapse (http://github.com/legalLab/protocols-scripts). We then generated a Bayesian Inference phylogeny using the software BEAST2 (Bouckaert et al., 2014) using the HKY+gamma model of molecular evolution (Hasegawa, Kishino & Yano, 1985); this model was chosen using the Akaike Information Criterion with correction (AICc) for model selection as implemented in the program jModelTest 2.1.10 (Posada, 2008). We ran two independent runs, generating 20 million topologies in each replicate. After checking for stationarity and convergence of the two chains in Tracer 1.7 (Rambaut et al., 2018), we combined the two runs, subsampled and burned-in the topologies to produce a final dataset of 3,000 topologies which we used to produce a maximum credibility tree in TREEANNOTATOR (Bouckaert et al., 2014).

We used the maximum credibility tree as input for three single locus species discovery analyses: GMYC, the general mixed Yule coalescent model (Fujisawa & Barraclough, 2013); bGMYC, a Bayesian implementation GMYC (Reid & Carstens, 2012); and mPTP, the multi-rate Poisson tree process method (Kapli et al., 2017). For GMYC, we used the package splits_1.0-19 (Fujisawa & Barraclough, 2013); for bGMYC, we used the package bGMYC 1.0.2 (Reid & Carstens, 2012). Since rooted phylograms are required input for mPTP, we optimized the topology, branch lengths, and gamma rate parameters of the maximum credibility tree using the optim.pml function of phangorn_2.3.1 (Schliep, 2011), all implemented in the R statistical software v3.4.1 (R Development Core Team, 2011). For mPTP analysis, we used the stand alone software mptp 0.2.3 (Kapli et al., 2017) using the optimized tree as input. Results were visualized using ggtree (Yu et al., 2017), also implemented in R.

#### Divergence-time estimates

Following single locus species discovery analyses, we chose the most frequent haplotype from each lineage discovered in the GMYC analysis and combined these haplotypes with outgroup sequences from *Alligator mississippiensis* (EU496863), *A. sinensis* (AF432918), *Melanosuchus niger* (EU161675), *Caiman latirostris* (EU161674), *C. yacare* (JF315314), *C. crocodilus* (EU161660) and *Paleosuchus palpebrosus* (MH846344, MH846457, MH846503) (Glenn et al., 2002; Hrbek et al., 2008; Oaks, 2011; Muniz et al., 2018). We inferred the divergence time between main lineages of *Paleosuchus trigonatus and P. palpebrosus* using the add-on package CladeAge (Matschiner et al., 2017) implemented in the Bayesian software BEAST 2 (Bouckaert et al., 2014). We also used the package bModelTest (Bouckaert & Drummond, 2017) to estimate the best site-substitution model.

For divergence-time estimation, we used the lognormal relaxed molecular clock (Drummond et al., 2006), the birth-death tree prior (Gernhard, 2008) with default priors for the birth rate and the relative death rate, and a random starting tree with a root height value of 90 Ma, older than the currently accepted divergence between Alligatorinae-Caimaninae (71–66 Ma) (Müller & Reisz, 2005). Following Bona et al. (2018), we set four fossil constraints as calibration points for the CladeAge model: †*Navajosuchus mooki* as the oldest known Alligatorinae, †*Brachychampsa sealeyi* as the oldest known Caimaninae, *Caiman cf. C.* †*brevirostris* as the oldest known *Caiman* and Jacarean, and *Paleosuchus* sp. as the oldest known *Paleosuchus* fossil record. For the CladeAge net diversification rate parameter, we used the minimum (0.006007672) and maximum (0.023543042) values for Alligatoridae diversification rate estimated by Scholl & Wiens (2016), and default values for the turnover rate and sampling rate parameters. Fossil age ranges, CladeAge parameters, and its references are listed in Table 1.

**Table 1:** Divergence-time estimates (median and 95% HPD) within Alligatoridae crown group using fossil calibration points (highlighted in bold) and the Clade Age calibration method. Clade Age model parameters are listed below.

**Clade age calibration**					
**Node**	**Median**	**95% HPD**	**Fossil constraint**	**Age range**	**Reference**
1	90.72	71.61–127.59	–	–	–
**2**	**57.23**	**37.8–86.1**	*Navajosuchus mooki*	63.3–67.1	Mook (1942)
**3**	**60.08**	**42.09–86.3**	*Brachychampsa sealeyi*	70.3–84.9	Williamson (1996)
**4**	**28.23**	**18.12–42.89**	*Paleosuchus* sp.	11.8–13.8	Salas-Gismondi et al. (2015)
5	4.17	2.08–6.78	–	–	–
6	3.08	1.55–5.42	–	–	–
7	0.52	0.05–1.31	–	–	–
8	7.5	4.2–12.34	–	–	–
9	0.98	0.21–2.18	–	–	–
**10**	**37.36**	**24.5–54.64**	*Caiman* cf.*C. brevirostris*	17.21–18.27	Solórzano et al. (2018)
**11**	**22.13**	**13.88–33.27**	*Caiman* cf.*C. brevirostris*	17.21–18.27	Solórzano et al. (2018)
12	7.32	4.11–11.89	–	–	–

**Notes.**

Net diversification rate (min–max): 0.006007672–0.023543042 (Scholl & Wiens, 2016).

Turnover rate: default.

Sampling rate: default.

We ran three MCMC simulations of 20,000,000 generations, sampling tree topologies and parameters every 1,800 generations, discarding the first 10% generations as burn-in. We used the software Tracer 1.7 (Rambaut et al., 2018) to check convergence of chains and parameters by comparison of the ln likelihood values and effective sample size (ESS > 200).

### Population structure analysis

To explore the population genetic structure within *P. trigonatus* we used a Bayesian clustering analysis implemented by Corander, Waldmann & Sillanpää (2003) in the program BAPS 6.0. We used all individual sequences in the dataset, and a set of K values between 2 to 8 as *a priori* information for the clusters (assuming a total of eight drainages sampled), with 10 repetitions for each value of K. We assumed clusters with the lowest log (marginal likelihood) values and highest posterior probability to be the best fit for the data. We used Arlequin 3.5 (Excoffier & Lischer, 2010) to estimate number of haplotypes, segregating sites, haplotype and nucleotide diversity, Tajima’s D (Tajima, 1989), and Fu’s Fs (Fu, 1997) neutrality tests for each cluster.

We performed a Mantel test to test the correlation between genetic distances and geographic distances between the sampled localities (Mantel, 1967). In this analysis, we provided a matrix of Euclidean geographic distances and a matrix of pairwise Φ_*ST*_ values (Weir & Cockerham, 1984) to verify if the population structure could be explained as a function of geographic distance. We used a pairwise Φ_*ST*_ population structure between river drainages matrix as input for a non-metric multidimensional scaling analysis (MDS) (Kruskal, 1964) utilizing the isoMDS function of the MASS package (Venables & Ripley, 2002) in the software R (R Development Core Team, 2011). This analysis consists of representing in a K-dimensional space (defined a priori) an array of dissimilarities or relative distances generated from pairwise comparisons between objects. We used a value of *k* = 3 for the number of dimensions (after checking stress values and the Shepard diagram) and default settings of the function. We evaluated the goodness-of-fit by analyzing stress values, where values 0.05 < 0.2 are considered poor and <0.05 excellent. We also plotted the dissimilarity matrix between the drainage basins in a three-dimensional scatterplot created using the R package scatterplot3d (Ligges & Mächler, 2003).

In order to visualize the genealogical relationships of the Cytochrome *b* haplotypes, we generated a haplotype network in the software HAPLOVIEWER (Salzburger, Ewing & Von Haeseler, 2011). For the construction of the network, we used as input all *P. trigonatus* sequences and a maximum likelihood phylogeny of these sequences estimated in the program PhyML 3.2 (Guindon & Gascuel, 2003) under the TrN+I+G model of molecular evolution inferred in the program jModelTest 2.1.10 (Posada, 2008).

## Results

We obtained a total of 1,020 bp of the mitochondrial Cytochrome *b* gene (89.24%, 1,143 bp) after the alignment and manual edition of 230 samples of *P. trigonatus* from 42 localities. We observed a total of 36 haplotypes with 57 polymorphic sites, where 47 sites were parsimony informative and 10 singletons. Most frequent haplotypes were H8 (68/230), H7 (41/230), and H4 (32/230), representing 61.30% of the dataset. Uncorrected p-distance between haplotypes varied from 0.1 to 3.6%. All haplotypes, polymorphic sites, nucleotide position, and number of individuals per haplotype are listed in Table S2. Relationships between haplotypes was visualized as a haplotype network in Fig. 1.

**Figure 2:**
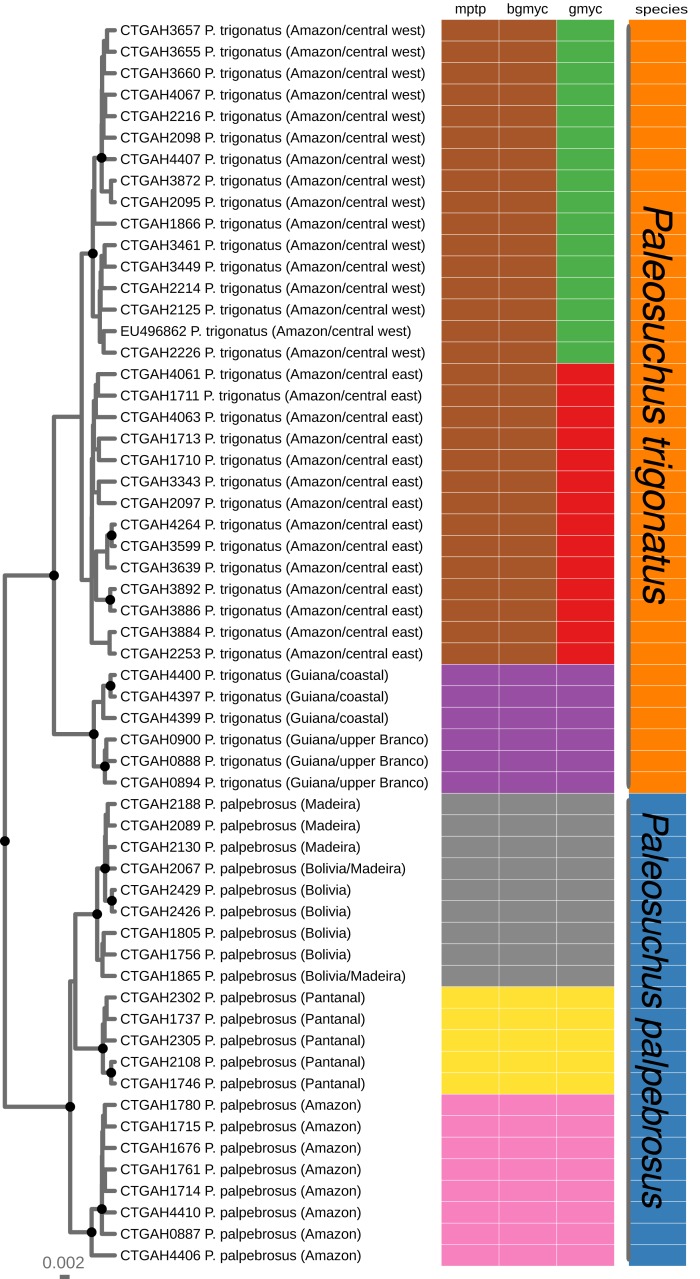
Maximum clade credibility chronogram generated using BEAST. Maximum clade credibility chronogram from 9,000 posterior trees generated using BEAST. Dataset comprised 36 unique haplotypes of *P. trigonatus*, and 22 unique haplotypes of *P. palpebrosus* (Muniz et al., 2018) cytb sequences (1020 aligned base pairs). Bayesian posterior probabilities above 0.95 are shown as dark nodes. MPTP and bGMYC single locus species discovery methods resulted in the discovery of five lineages, two of *P. trigonatus* and three of *P. palpebrosus* (see also Muniz et al., 2018). GMYC resulted in the discovery of six lineages, representing population structuring identified by other analyses carried out in this study.

### Single locus species discovery analyses

We performed three different single locus species discovery analyses. All three were congruent in identifying five principal lineages of *Paleosuchus*, three lineages of *P. palpebrosus* and two lineages of *P. trigonatus* (Fig. 2). GMYC additionally subdivided the Amazonian (ex. Branco River) lineage of *P. trigonatus* into two geographically structured clades east and west of the north-south flowing Negro and Madeira rivers. This division is also clearly evident in the haplotype network (Fig. 1). All three methods of single locus species discovery analyses recovered the three lineages of *P. palpebrosus* reported in Muniz et al. (2018).

### Phylogenetic analysis and divergence-time estimation

**Figure 3:**
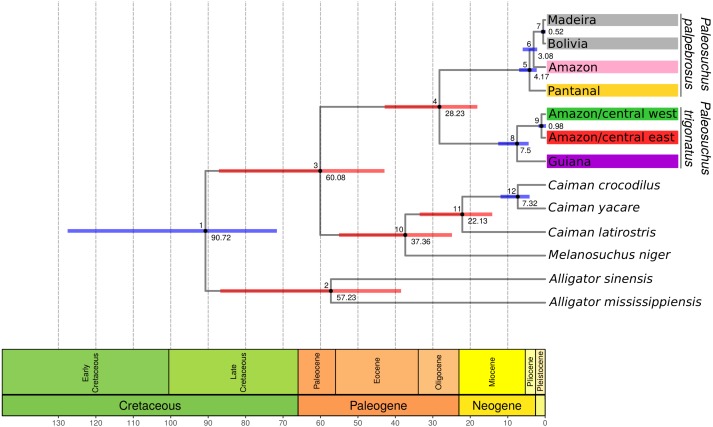
BEAST cytochrome b chronogram for *P. trigonatus* using the CladeAge calibration method. Numbers below nodes correspond to time in millions of years and bars represent 95% HPD intervals, where red bars indicates fossil constraints. The colors boxes represents the cluster to which the analyzed individuals belong based on species discovery assignments, indicating Amazonia lineage (red and green) and Guiana lineage (purple).

Bayesian timetree recovered a well supported phylogeny; with exception of support for the phylogenetic position of Amazon and Pantanal *P. palpebrosus,* all nodes were supported by posterior probability values of 1 (Fig. 3). The divergence between the Guiana and Amazonia lineages was estimated at 7.5 Ma (95% highest posterior density (HPD) = 4.20–12.34 Ma) (Fig. 3) while the divergence between the central-west and central-east clades of the Amazonia lineage was estimated at 0.98 Ma (95% HPD = 0.21–2.18 Ma). In *Paleosuchus palpebrosus* the basal divergence occurred at 4.17 Ma (95% HPD = 2.08–6.78 Ma), while divergence between the Bolivia/Madeira and Amazon/Pantanal lineages was estimated at 3.08 Ma (95% HPD = 1.55–5.42 Ma). The median and 95% HPD intervals of divergence time estimates are summarized in Table 1.

### Population structure analysis

BAPS clustering method recovered *K* = 4 as the optimal number of clusters of *Paleosuchus trigonatus* (logML = −1002.4532, *p* = 1) (Fig. 1). Within the Guiana lineage, the analysis recovered two structured populations with no shared haplotypes: one from Atlantic coast drainages and another from the headwaters and tributaries of the upper Branco River. The Amazonia lineage was also composed of two populations: a central-west cluster and a central-east cluster. While populations at the extremes of the distribution sampled for the Amazonia lineage were composed entirely of haplotypes of either one or the other cluster, central Amazonia populations comprised individuals with haplotypes from both clusters (Fig. 1).

Transition between the Guiana and Amazonia lineages occurs in the middle Branco River region, with individuals in the northern section of this region belonging to the Guiana lineage and southern individuals to the Amazonia lineage. We failed to detect co-occurrence of these lineages in any given locality. Of the 43 individuals sampled in the lower Negro River drainages, only four individuals of the central-west cluster were found. In contrast, lower Purus River drainages had only one individual from central-east cluster. Madeira River basin had the most heterogeneous haplotype composition. Upper portions of the Madeira River were almost entirely comprised of central-west haplotypes, whereas the contribution of these haplotypes decreased in downstream localities of the Madeira.

General genetic parameters of *Paleosuchus trigonatus* in each genetic cluster are shown in the Table 2. *Paleosuchus trigonatus* shows high levels of haplotype (0.856 ± 0.015) and nucleotide diversity (0.0119 ± 0.001) considering all samples simultaneously. Analyzing individuals from each cluster separately, the lowest haplotype diversity was found in the upper Branco River (0.116 ± 0.073), and the highest in the central-west cluster (0.748 ± 0.043). This pattern is paralleled by number of polymorphic loci, number of haplotypes and nucleotide diversity. Tajima’s *D* was significant for Coastal (−1.6670), upper Branco (−1.7282), and central-east (−1.6848) clusters, while Fu’s *Fs* was significant for upper Branco (−1.746), central-east (−10.680), and central-west (−8.669) clusters. Both Coastal and upper Branco had the lowest Nei’s haplotype diversity and smallest number of haplotypes.

**Table 2:** General genetic parameters of *P aleosuchus trigonatus* genetic clusters. N = sample size; S = segregating sites (polymorphic); Hap = number of haplotypes; H = Nei’s haplotype diversity; pi = Nei’s nucleotide diversity; D = Tajima’s *D* with simulated *p*-value (alpha = 0.05, 10,000 simulations); Fu’s *Fs* with simulated *p*-value (alpha < 0.02, 10,000 simulations).

**Cluster**	**N**	**S**	**Hap**	**H**	**pi**	***D*(p)**	***Fs*(p)**
Coastal	10	4	3	0.377 ± 0.181	0.00078 ± 0.0006	**−1.6670 (0.018)**	0.058 (0.411)
Upper Branco	34	3	3	0.116 ± 0.073	0.00017 ± 0.0002	**−1.7267 (0.011)**	**−1.785 (0.017)**
Central East	99	12	14	0.520 ± 0.060	0.00083 ± 0.0006	**−1.6848 (0.021)**	**−10.680 (<0.0001)**
Central West	87	14	16	0.748 ± 0.043	0.00147 ± 0.001	−1.2821 (0.084)	**−8.669 (0.01)**
Total	230	57	36	0.856 ± 0.015	0.0119 ± 0.001	0.8360 (0.100)	0.402 (0.63)

The result of the Mantel test was not significant, that is, there was no significant correlation between matrices of pairwise Φ_*ST*_ and matrices of Euclidean geographic distance between the sampled localities (*r* = 0.199739, *p* = 0.101900). Pairwise Φ_*ST*_ shows a very high degree of population structuring between river drainages. All comparisons between the Coastal or upper Branco River and other river drainages show Φ_*ST*_ values greater than 0.9 (Table 3).

**Table 3:** Pairwise Φ_*ST*_ values between river drainages sampled in this study. Significant values are in bold (*p* < 0.0003 after Bonferroni correction (Rice, 1989)).

Φ_*ST*_	**Coastal**	**Upper** **Branco**	**Lower** **Negro**	**Lower** **Purus**	**Aripuana**	**Madeira**	**Upper** **Madeira**
Coastal	–						
Upper Branco	**0.9234**	–					
Lower Negro	**0.9670**	**0.9765**	–				
Lower Purus	**0.9686**	**0.9824**	**0.7080**	–			
Aripuana	**0.9577**	**0.9805**	0.0991	**0.6737**	–		
Madeira	**0.9221**	**0.9420**	**0.3364**	0.1563	**0.2862**	–	
Upper Madeira	**0.9592**	**0.9795**	**0.6703**	0.1167	**0.6045**	0.1047	–
Xingu	**0.9646**	**0.9793**	**0.1453**	**0.7395**	0.1406	**0.3922**	**0.6940**

**Figure 4:**
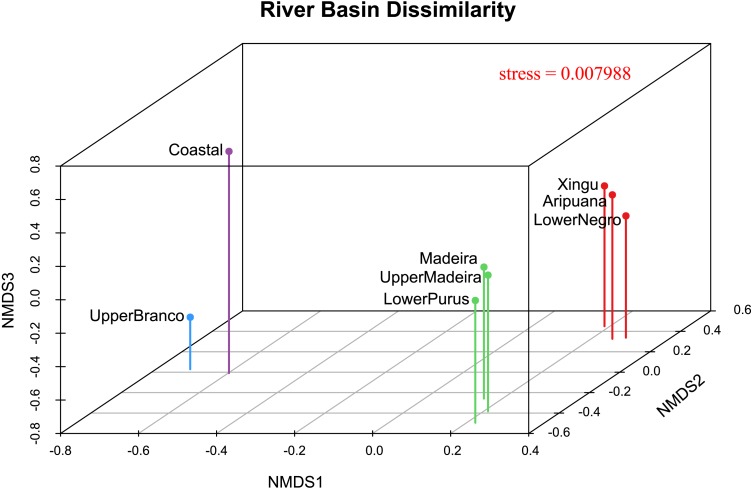
River basin dissimilarity scatter plot based on a pairwise Φ_*ST*_ genetic distance matrix between populations of *P. trigonatus* using a non-metric multidimensional scaling analysis (MDS). Stress value indicates goodness-of-fit. The analysis was made utilizing the isoMDS function of the MASS package (Venables & Ripley, 2002) and the scatter plot was made using scatterplot3d (Ligges & Mächler, 2003) in the software R (R Development Core Team, 2011). The colors represents the cluster to which the analyzed individuals belong based on BAPS assignments, indicating the Amazonia lineage (red and green) and the Guiana lineage (purple and blue).

The 3D plot of the MDS axes shows, in the first axis (NMDS1), a clear dissimilarity between the Coastal or upper Branco River and all other river drainages (Fig. 4). The second axis (NMDS2) clearly separates the upper Madeira, Madeira and lower Purus from the Xingu, Aripuanã and lower Negro. It is important to note that the Aripuanã is one of the tributaries of the right bank of the Madeira River but presents a great dissimilarity in relation to the Madeira River. The third axis (NMDS3) separates the Coastal and upper Branco basins.

## Discussion

The cytochrome *b* gene genealogies of *Paleosuchus trigonatus* show clear evidence of evolutionarily independent lineages. Formal species discovery analyses identified five principal groups of *Paleosuchus*, three lineages of *P. palpebrosus* and two lineages of *P. trigonatus*. With exception of the phylogenetic support for the position of Amazon and Pantanal *P. palpebrosus,* posterior probabilities of all lineages are strongly supported. The GMYC single locus species discovery method discovered additional groups which correspond to substructure within the Amazonia lineage; also evident in haplotype network, BAPS clustering and MDS analyses. Fossil calibration recovered the divergence time between the Guiana and Amazonia lineages of 7.5 Ma, situated at the Miocene/Pliocene boundary (Fig. 3).

Based on current fossil record, the number of crocodilian genera decreased from 26 to nine in the Miocene and Pleistocene (Markwick, 1998; Brochu, 2003; Scheyer et al., 2013). In the Amazon, for example, the Neogene (23.03–2.58 Ma) was marked by great geological and climatic changes, such as the terminal state of Andean orogeny, which led to the breaching of the Purus Arch, the transition from a large lake/wetland system in the western Amazon (Lake Pebas) to a river system, and the formation of the modern Amazon River (Hoorn et al., 2010). This event is thought to have lead to the extinction of a rich South American crocodilian fauna (Riff et al., 2009; Salas-Gismondi et al., 2015; Moreno-Bernal, Head & Jaramillo, 2016). One of the hypotheses for the preferential survival of taxa is their geographic distribution (Jablonski, 1987); species, including crocodilians, with broad geographic distributions are more likely to survive than species with restricted geographic distribution (Markwick, 1998). The surviving species would then diversify although this may not necessarily be evident in the morphology of these groups (Oaks, 2011; Shirley et al., 2014; Muniz et al., 2018). This pattern is increasingly evident in phylogenetic analyses using molecular markers (Venegas-Anaya et al., 2008; Eaton et al., 2009; Hekkala et al., 2011; Shirley et al., 2014; Bloor, Ibáñez & Viloria-Lagares, 2015; Milián-García et al., 2017; Muniz et al., 2018).

Our results recovered a median divergence-time of *P. trigonatus* and *P. palpebrosus* at 28.23 Ma, with 95% HPD intervals between 42.89 to 18.12 Ma (Fig. 3; Table 1, node 4), and the Alligatorinae-Caimaninae divergence at 90.72 Ma, with 95% HPD intervals between 127.59 to 71.68 Ma (Fig. 3; Table 1, node 1). Previous molecular calibrations recovered divergence-times between 71 to 50.55 Ma for the Alligatorinae-Caimaninae divergence (Roos, Aggarwal & Janke, 2007; Oaks, 2011; Shirley et al., 2014) and the *P. trigonatus*-*P. palpebrosus* divergence at 19 to 17 Ma using mitogenomes (Roos, Aggarwal & Janke, 2007), 13.64 to 7.54 Ma using species trees (Oaks, 2011), and 16.67 to 8.85 Ma using species trees or 11.19 to 0.99 Ma using only nuDNA dataset (Shirley et al., 2014).

All previous studies relied on the Alligatorinae-Caimaninae divergence (71 to 66 Ma) of Müller & Reisz (2005) as a fossil constraint, which was based on the sister taxon relationship between †*Strangerochampsa mccabei* and alligatorids recovered by Brochu (1999). However, the results of Bona et al. (2018) indicates that the earliest caimanines had a North American origin and some of the taxa previously considered alligatoroids, such as †*Strangerochampsa mccabei,* †*Brachychampsa,* and †*Albertochampsa,* would be the most basal caimanines, pushing the Alligatorinae-Caimaninae divergence further back into the Cretaceous (100–90 Ma). Thus, molecular calibrations such as from Oaks (2011), which constrained the Alligatorinae-Caimaninae divergence at 71 to 64 Ma and the Crocodylidae-Alligatoridae origin at 100 or 90 Ma should be at least revisited if Bona et al. (2018) turns out to be a paradigm shift in the early evolutionary history of Caimaninae. Independent of the phylogenetic reconstruction or calibration methods, the divergence of *P. palpebrosus* and *P. trigonatus* estimated from mtDNA datasets is always much older than that estimated from nuDNA. Although at this point speculative, this might be due to introgressive hybridization of diverging lineages marked by their mtDNA genomes as has been observed between *P. palpebrosus* lineages (Muniz et al., 2018) and *Inia* dolphins (Gravena et al., 2015). Although mitochondrial lineages potentially count only part of the evolutionary history of the species, this history is as pertinent as the evolutionary history of the nuclear genome. Furthermore, conflicts, if any, between these histories reflect an added layer of evolutionary complexity beyond simple populations/species divergence. Thus, all histories are relevant and important for the understanding of an organism’s evolutionary history.

The divergence-time between the Guiana and Amazonia lineages of *P. trigonatus* was estimated at 7.5 Ma (Fig. 3; Table 1, node 8), at the Miocene/Pliocene boundary. Lineage diversification of other species of Caimaninae also dates to the Pliocene and Pleistocene, such as those of the species of *Caiman* and subspecies of *Caiman crocodilus* (Venegas-Anaya et al., 2008) and lineages of *Paleosuchus palpebrosus* (Fig. 3; Table 1, nodes 5 and 6), a trend present in most of South American herpetofauna (Turchetto-Zolet et al., 2012), often linked to major tectonic and palaeogeographical reorganizations (Neogene) or environmental shifts caused by the glacial-interglacial periods (Quaternary) (Rull, 2011). Although it is unclear which events lead to the divergence of the Guiana –Amazonia lineages of *P. trigonatus*, Atlantic coast drainages might have been colonized by populations of the southeast or central Amazon where the H8 haplotype occurs, and which likely was the ancestral haplotype or shares a most recent common ancestor with a haplotype from which the Guiana lineage is derived (Fig. 1). Further support for this hypothesis comes from the relatively low genetic diversity of these populations when compared to the Amazon basin populations and the signature of population expansion found in Tajima’s D (*D*_Coastal_ =  − 1.667, *p* = 0.018; *D*_Branco_ =  − 1.7282, *p* = 0.011) and Fu’s Fs (*Fs*_Branco_ =  − 1.785, *p* = 0.017). Future studies using both mitochondrial and nuclear DNA genomes will allow us to estimate which processes (e.g., vicariance vs dispersal) favored this divergence, which routes the ancestors might have used to colonize the Amazon and Atlantic coast drainages, and to which extent their distributions overlap.

Our results already indicate that a transition zone between the Guiana and Amazonian lineages exists in the region of the middle Branco River. There is evidence that the upper Branco River, the main tributary of the Negro River, flowed SW–NE towards the Caribbean Sea, integrating the Proto-Berbice basin, one of the largest drainage basins in the central portion of the Guiana Shield, during the Late Neogene or Early Quaternary (5.3–0.8 Ma) (Schaefer & Dalrymple, 1996; Cremon et al., 2016). This basin would have been physically disconnected from the Amazon basin due to the existence of highlands and a chain of mountains called the “paleodivisor” Amazon/Proto–Berbice (Fig. 1, orange highlighted area), which were eroded by the intensive erosion regime associated with arid and semi–arid climates, initiated in the Oligocene, culminating in its complete destruction in Plio/Pleistocene (1.8–1.0 Ma) (Schaefer & Vale Júnior, 1997).

Although Cremon et al. (2016) find that ”there is an overall lack of geological and geomorphological data to test [either the paleodivisor hypothesis or any hypothesis based on erosion of highland basement rocks of the Guiana Shield, allowing the connection between adjacent basins]” (page 23, first paragraph), the authors provide evidence that tectonic and climactic events had a major role in reordering and capturing the drainage of the upper Branco River by the Negro River, an event that would have occurred at least 18.7 Ka in the Late Pleistocene. Other evidence for the existence of the Proto-Berbice basin is the co-occurrence of fish species in the Branco and Essequibo river basins (Lujan, 2008; Lujan & Armbruster, 2011; De Souza, Armbruster & Werneke, 2012). In fact, the Takutu River, the main eastern tributary of the Branco River, and the Rupununi River, a tributary of the Essequibo River, share 254 freshwater fish species, that is ∼73.8% of the total sampled species (De Souza, Armbruster & Werneke, 2012). Furthermore, there is also evidence of recent connection between fish populations/lineages from the upper Branco River and the Orinoco via the Essequibo (see Escobar et al., 2015 and references within).

Considering the above scenario, it is possible to infer that the most likely colonization route for the former Proto-Berbice basin was via the northern Atlantic coast rivers. Populations of the Proto-Berbice would be disconnected from populations of the middle and lower reaches of the Branco and Negro rivers (either by the existence of the paleodivisor or due the lack of a dispersal corridor between these areas) until the drainage capture of some of the former Proto-Berbice headwaters (which currently are Branco river headwaters) by the present-day middle and lower sections of the Branco River. This capture event changed the drainage flow in the southward direction, connecting the upper Branco River with the Negro River, and disconnecting the upper Branco from the Proto-Berbice. These events would explain the co-occurence of both lineages (Amazonia and Guiana) in the middle Branco River and the structure between Branco and Atlantic coast populations. Connections between Amazon basin and coastal river drainages were also claimed in order to explain the unexpected structuring of genetic diversity in the catfish *Pseudoancistrus brevispinis* (Cardoso & Montoya-Burgos, 2009), suggesting a single colonization event from the Amazon basin towards the headwaters of northern Atlantic coastal rivers or via coastal marshes.

The Amazon basin lineage is structured along an east–west gradient, with a sharp transition in haplotype group frequencies to the east and west of the Negro and Madeira rivers. This pattern is also observed in other groups of reptiles (e.g., Glor, Vitt & Larson, 2001; Kronauer et al., 2005; Gamble et al., 2008), amphibians (Simula, Schulte & Summers, 2003), and caimans (Vasconcelos et al., 2006; Vasconcelos et al., 2008). Vasconcelos et al. (2006) found significant genetic structure between *Caiman crocodilus* sampled in localities of the Amazon basin and Atlantic coast drainages paralleling the pattern found in *P. trigonatus*. While isolation-by-distance was discarded by the authors for *C. crocodilus*, it appears to have been an important structuring factor for *Melanosuchus niger* populations sampled across the Amazon basin and Atlantic coast drainages (Vasconcelos et al., 2008) paralleling the east–west divergence observed in *P. trigonatus*.

We also observed that the Aripuanã River is structured with respect to the Purus and Madeira, its *P. trigonatus* being most similar to *P. trigonatus* of the Negro River basin, although the Aripuanã is one of the tributaries of the right margin of the Madeira River. This biological connection between the Aripuanã and Negro river basins is intriguing, but has been observed in fishes as well (e.g., Amado, Hrbek & Farias, 2011; Collins et al., 2018; Machado et al., 2018), suggesting a recent connection of these two basins.

In general, drainage samples from the Brazilian and southwestern Guiana Shields (Aripuanã, Jaci-Paraná, Manicoré, Juruena, Xingu, Negro, Uatumã) present, in great majority, haplotypes of the central-eastern cluster detected by BAPS (see Fig. 1). On the other hand, samples collected in the upper course of Madeira River channel (upper Madeira, Madeira between rapids, Porto Velho and Humaitá) presented a mixture of haplotypes, most of them from the central-west cluster. This heterogeneity of the Madeira River basin gives further credence to it being a conglomerate basin (Dagosta & Pinna, 2017).

In conclusion, most of the individuals of *P. trigonatus* of Amazon basin rivers draining the Brazilian and Guiana Shields have central-eastern haplotypes, while rivers of Andean origin have central-western haplotypes. As the Madeira River basin receives input from both the Andes and the Brazilian Shield, this would explain the greater admixture proportion of the central-eastern and central-western haplotype groups in the Jaci-Paraná, Manicoré and Aripuanã rivers.

### Implications for conservation

This study provides evidence for the existence of two divergent lineages of *P. trigonatus*, providing yet another example of species with a broad geographic distribution and considered at low conservation risk by the IUCN, that comprise multiple independent lineages. These findings have an important bearing on the future of conservation of *P. trigonatus*. Lineages and/or cryptic species often are under anthropogenic threat or otherwise conservation dependent, something that would not be evident without the recognition of these lineages are evolutionarily independent entities (Shirley et al., 2014). Although the two lineages diverged at the Miocene/Pleistocene boundary, we acknowledge that additional data, such as genomics, morphometrics and ecology will strengthen the inference that these lineages likely are different species.

One of the major problems for the taxonomic revision of *P. trigonatus* is uncertainly about its type locality. Older revisions (e.g., Schmidt, 1928; Mook & Mook, 1940) did not included any information about the type locality or stated it as “unknown” (e.g., Gray, 1862; King & Burke, 1989; Magnusson, 1992). Bauer & Günther (2006) state that both the jar label and catalogue entry of the type specimen of *Crocodilus trigonatus* Schneider, 1801 (=*Paleosuchus trigonatus*)—a specimen from the private collection of Marcus Elieser Bloch—gives the locality “Süd America”.

The problem, therefore, is to which lineage/species does the type specimen belongs to, and thus which of these two lineages is *Paleosuchus trigonatus*. Since it is probably impossible to track down the type locality of the ZMB 243 specimen based on historical data, we suggest another approach. By the use of ancient DNA techniques (Pääbo, Higuchi & Wilson, 1989) it would be possible to assign to which lineage the holotype of *P. trigonatus* belongs to. Additionally, assuming two premises: (1) specimens from the type locality or its descendants still exist, and (2) that the lineages are geographically structured, it would be possible to assign the holotype to its topotypical populations with high accuracy by screening several loci (SNPs) generated by Next Generation Sequencing techniques and analyzed by methods such as the Discrimant Analysis of Principal Components (DAPC) (Jombart, Devillard & Balloux, 2010).

## Conclusions

Geographically broadly distributed species are in particular need of studies that investigate population structuring and the existence of cryptic species hidden under one binomial epithet. It is only such analyses that will provide solid scientific data on diversity and distribution of these lineages, which, ultimately, underpin conservation efforts and management guidelines. This study provides evidence for the existence of two deeply divergent lineages of *Paleosuchus trigonatus*—Guiana and Amazonia—both strongly supported by species discovery, BAPS clustering and MDS analyses, with the two lineages diverging at the Miocene/Pliocene boundary. The Guiana lineage occupies Atlantic coastal draining rivers, and the upper reaches of the Branco River—an Amazon basin river—upstream of the Caracarai rapids, indicating a recent capture of the upper Branco River by the lower Branco. Within the Amazon basin, haplotype frequencies along the east–west axis sharply transition at the Negro and Madeira rivers, a pattern commonly observed not only in aquatic taxa but also in varzea birds (Cohn-Haft, Naka & Fernandes, 2007). This study, therefore, is an important contribution to understanding the evolution of Amazonian biota in general, and for the conservation of *P. trigonatus* and its lineages in particular.

##  Supplemental Information

10.7717/peerj.6580/supp-1 Table S1 Sampling localities and number of samples (N) of *Paleosuchus trigonatus* utilized in this study

10.7717/peerj.6580/supp-2 Table S2 Haplotypes (H), polymorphic sites, nucleotide position, and number of individuals per haplotype (N) sampled in this study

## Additional Information and Declarations

### Data Availability

The following information was supplied regarding data availability: GenBank: MH757465–MH757694.

## References

[ref-1] Amado MV, Hrbek T, Farias IP (2011). A molecular perspective on systematics, taxonomy and classification Amazonian discus fishes of the genus *Symphysodon*. International Journal of Evolutionary Biology.

[ref-2] Bauer AM, Günther R (2006). An annotated catalogue of the types of crocodilians (Reptilia: Crocodylia) in the collection of the Museum für Naturkunde der Humboldt-Universität zu Berlin (ZMB). Zoologische Reihe.

[ref-3] Bloor P, Ibáñez C, Viloria-Lagares TA (2015). Mitochondrial DNA analysis reveals hidden genetic diversity in captive populations of the threatened American crocodile (*Crocodylus acutus*) in Colombia. Ecology and Evolution.

[ref-4] Bona P, Ezcurra MD, Barrios F, Fernandez Blanco MV (2018). A new Palaeocene crocodylian from southern Argentina sheds light on the early history of caimanines. Proceedings of the Royal Society B: Biological Sciences.

[ref-5] Bouckaert RR, Drummond AJ (2017). bModelTest: bayesian phylogenetic site model averaging and model comparison. BMC Evolutionary Biology.

[ref-6] Bouckaert R, Heled J, Kühnert D, Vaughan T, Wu C, Xie D, Suchard MA, Rambaut A, Drummond AJ (2014). BEAST 2: a software platform for bayesian evolutionary analysis. PLOS Computational Biology.

[ref-7] Brochu CA (1999). Phylogenetics, taxonomy, and historical biogeography of alligatoroidea. Journal of Vertebrate Paleontology.

[ref-8] Brochu CA (2003). Phylogenetic approaches toward crocodylian history. Annual Review of Earth and Planetary Sciences.

[ref-9] Brochu CA (2007). Morphology, relationships, and biogeographical significance of an extinct horned crocodile (Crocodylia, Crocodylidae) from the Quaternary of Madagascar. Zoological Journal of the Linnean Society.

[ref-10] Campos Z, Mourão G, Magnusson WE (2017). The effect of dam construction on the movement of dwarf caimans, *Paleosuchus trigonatus* and *Paleosuchus palpebrosus*, in Brazilian Amazonia. PLOS ONE.

[ref-11] Campos Z, Muniz F, Magnusson WE (2012). Dead *Paleosuchus* on roads in Brazil. Crocodile Specialist Group Newsletter.

[ref-12] Campos Z, Muniz F, Magnusson WE (2017). Extension of the geographical distribution of Schneider’s Dwarf Caiman, *Paleosuchus trigonatus* (Schneider, 1801) (Crocodylia: Alligatoridae), in the Amazon-Cerrado transition, Brazil. Check List.

[ref-13] Cardoso YP, Montoya-Burgos JI (2009). Unexpected diversity in the catfish *Pseudancistrus brevispinis* reveals dispersal routes in a Neotropical center of endemism: The Guyanas Region. Molecular Ecology.

[ref-14] Castresana J (2001). Cytochrome b phylogeny and the taxonomy of great apes and mammals. Molecular Biology and Evolution.

[ref-15] Cohn-Haft M, Naka LN, Fernandes AM (2007). Padrões de distribuição da avifauna da várzea dos rios Solimões e Amazonas. Conservação da Várzea: Identificação e Caracterização de Regiões Biogeográficas.

[ref-16] Collins RA, Bifi AG, De Oliveira RR, Ribeiro ED, Py-Daniel LHR, Hrbek T (2018). Biogeography and species delimitation of the rheophilic suckermouth-catfish genus *Pseudolithoxus* (Siluriformes: Loricariidae), with the description of a new species from the Brazilian Amazon. Systematics and Biodiversity.

[ref-17] Corander J, Waldmann P, Sillanpää MJ (2003). Bayesian analysis of genetic differentiation between populations. Genetics.

[ref-18] Cremon ÉH, Rossetti DF, Sawakuchi AO, Cohen MCL (2016). The role of tectonics and climate in the late Quaternary evolution of a northern Amazonian River. Geomorphology.

[ref-19] Crocodile Specialist Group (1996). http://dx.doi.org/10.2305/IUCN.UK.1996.RLTS.T46588A11063247.en.

[ref-20] Dagosta FCP, Pinna M de (2017). Biogeography of Amazonian fishes: deconstructing river basins as biogeographic units. Neotropical Ichthyology.

[ref-21] De Souza LS, Armbruster JW, Werneke DC (2012). The influence of the Rupununi portal on distribution of freshwater fish in the Rupununi district, Guyana. Cybium.

[ref-22] Doyle JJ, Doyle JL (1990). Isolation of plant DNA from fresh tissue. Focus.

[ref-23] Drummond AJ, Ho SYW, Phillips MJ, Rambaut A (2006). Relaxed phylogenetics and dating with confidence. PLOS Biology.

[ref-24] Eaton MJ, Martin A, Thorbjarnarson J, Amato G (2009). Species-level diversification of African dwarf crocodiles (Genus *Osteolaemus*): a geographic and phylogenetic perspective. Molecular Phylogenetis and Evolution.

[ref-25] Escobar MDL, Andrade-López J, Farias IP, Hrbek T (2015). Delimiting evolutionarily significant units of the fish, *Piaractus brachypomus* (Characiformes: Serrasalmidae), from the Orinoco and Amazon River Basins with Insight on Routes of Historical Connectivity. Journal of Heredity.

[ref-26] Excoffier L, Lischer HEL (2010). Arlequin suite ver 3.5: a new series of programs to perform population genetics analyses under Linux and Windows. Molecular Ecology Resources.

[ref-27] Fennessy J, Bidon T, Reuss F, Kumar V, Elkan P, Nilsson MA, Vamberger M, Fritz U, Janke A (2016). Multi-locus analyses reveal four giraffe species instead of one. Current Biology.

[ref-28] Fu YX (1997). Statistical tests of neutrality of mutations against population growth, hitchhiking and background selection. Genetics.

[ref-29] Fujisawa T, Barraclough TG (2013). Delimiting species using single-locus data and the generalized mixed Yule coalescent approach: a revised method and evaluation on simulated data sets. Systematic Biology.

[ref-30] Futuyma DJ, Agrawal AA (2009). Macroevolution and the biological diversity of plants and herbivores. Proceedings of the National Academy of Sciences of the United States of America.

[ref-31] Gamble T, Bauer AM, Greenbaum E, Jackman TR (2008). Evidence of Gondwanan vicariance in an ancient clade of geckos. Journal of Biogeography.

[ref-32] Gernhard T (2008). The conditioned reconstructed process. Journal of Theoretical Biology.

[ref-33] Glenn TC, Staton JL, Vu AT, Davis LM, Bremer JRA, Rhodes WE, Brisbin IL, Sawyer RH (2002). Low mitochondrial DNA variation among American alligators and a novel non-coding region in crocodilians. Journal of Experimental Zoology.

[ref-34] Glor RE, Vitt LJ, Larson A (2001). A molecular phylogenetic analysis of diversification in Amazonian *Anolis* lizards. Molecular Ecology.

[ref-35] Gravena W, Da Silva VMF, Da Silva MNF, Farias IP, Hrbek T (2015). Living between rapids: genetic structure and hybridization in botos (Cetacea: Iniidae: *Inia* spp.) of the Madeira River, Brazil. Biological Journal of the Linnean Society.

[ref-36] Gray JE (1862). A synopsis of the species of alligators. Annals and Magazine of Natural History.

[ref-37] Guindon S, Gascuel O (2003). A simple, fast, and accurate algorithm to estimate large phylogenies by Maximum Likelihood. Systematic Biology.

[ref-38] Hasegawa M, Kishino H, Yano T (1985). Dating of the human-ape splitting by a molecular clock of mitochondrial DNA. Journal of Molecular Evolution.

[ref-39] Hekkala E, Shirley MH, Amato G, Austin JD, Charter S, Thorbjarnarson J, Vliet KA, Houck ML, Desalle R, Blum MJ (2011). An ancient icon reveals new mysteries: mummy DNA resurrects a cryptic species within the Nile crocodile. Molecular Ecology.

[ref-40] Hoorn C, Wesselingh FP, ter Steege H, Bermudez MA, Mora A, Sevink J, Sanmartín I, Sanchez-Meseguer A, Anderson CL, Figueiredo JP, Jaramillo C, Riff D, Negri FR, Hooghiemstra H, Lundberg J, Stadler T, Särkinen T, Antonelli A (2010). Amazonia through time: andean uplift, climate change, landscape evolution, and biodiversity. Science.

[ref-41] Hrbek T, Da Silva VMF, Dutra N, Gravena W, Martin AR, Farias IP (2014). A new species of river dolphin from Brazil or: How little do we know our biodiversity. PLOS ONE.

[ref-42] Hrbek T, Vasconcelos WR, Rebelo GH, Farias IP (2008). Phylogenetic relationships of South American Alligatorids and the *Caiman* of Madeira River. Journal of Experimental Zoology.

[ref-43] Jablonski D (1987). Heritability at the species level: analysis of geographic ranges of cretaceous mollusks. Science.

[ref-44] Jombart T, Devillard S, Balloux F (2010). Discriminant analysis of principal components: a new method for the analysis of genetically structured populations. BMC Genetics.

[ref-45] Kapli P, Lutteropp S, Zhang J, Kobert K, Pavlidis P, Stamatakis A, Flouri T (2017). Multi-rate Poisson tree processes for single-locus species delimitation under maximum likelihood and Markov chain Monte Carlo. Bioinformatics.

[ref-46] Kearse M, Moir R, Wilson A, Stones-Havas S, Cheung M, Sturrock S, Buxton S, Cooper A, Markowitz S, Duran C, Thierer T, Ashton B, Mentjies P, Drummond A (2012). Geneious basic: an integrated and extendable desktop software platform for the organization and analysis of sequence data. Bioinformatics.

[ref-47] King FW, Burke RL (1989). Crocodilian, tuatara and turtle species of the world. A taxonomic and geographic reference.

[ref-48] Kronauer DJC, Bergmann PJ, Mercer JM, Russell AP (2005). A phylogeographically distinct and deep divergence in the widespread Neotropical turnip-tailed gecko, *Thecadactylus rapicauda*. Molecular Phylogenetics and Evolution.

[ref-49] Kruskal JB (1964). Multidimensional scaling by optimizing goodness of fit to a nonmetric hypothesis. Psychometrika.

[ref-50] Ligges U, Mächler M (2003). Scatterplot3d - an R package for visualizing multivariate data. Journal of Statistical Software.

[ref-51] Lujan NK (2008). Description of a new *Lithoxus* (Siluriformes: Loricariidae) from the Guayana Highlands with a discussion of Guiana Shield biogeography. Neotropical Ichthyology.

[ref-52] Lujan NK, Armbruster JW, Albert JS, Reis RE (2011). The Guiana Shield. Historical biogeography of neotropical freshwater fishes.

[ref-53] Machado VN, Collins RA, Ota RP, Andrade MC, Farias IP, Hrbek T (2018). One thousand DNA barcodes of piranhas and pacus reveal geographic structure and unrecognised diversity in the Amazon. Scientific Reports.

[ref-54] Magnusson WE, Hall P, Bryant R (1989). Paleosuchus. Crocodiles: their ecology, management and conservation.

[ref-55] Magnusson WE (1992). Paleosuchus trigonatus. Catalogue of American Amphibians and Reptiles.

[ref-56] Magnusson WE, Campos Z, Manolis SC, Stevenson C (2010). Schneider’s smooth-fronted caiman *Paleosuchus trigonatus*. Crocodiles: status survey and conservation action plan.

[ref-57] Magnusson WE, Lima AP (1991). The ecology of a cryptic predator, *Paleosuchus trigonatus*, in a tropical rainforest. Journal of Herpetology.

[ref-58] Mantel N (1967). The detection of disease clustering and a generalized regression approach. Cancer Research.

[ref-59] Markwick PJ (1998). Fossil crocodilians as indicators of Late Cretaceous and Cenozoic climates: implications for using palaeontological data in reconstructing palaeoclimate. Palaeogeography, Palaeoclimatology, Palaeoecology.

[ref-60] Matschiner M, Musilová Z, Barth JMI, Starostová Z, Salzburger W, Steel M, Bouckaert R (2017). Bayesian phylogenetic estimation of clade ages supports trans-Atlantic dispersal of cichlid fishes. Systematic Biology.

[ref-61] Meyer A (1994). Shortcomings of the cytochrome b gene as a molecular marker. Trends in Ecology & Evolution.

[ref-62] Milián-García Y, Castellanos-Labarcena J, Russello MA, Amato G (2017). Mitogenomic investigation reveals a cryptic lineage of *Crocodylus* in Cuba. Bulletin of Marine Science.

[ref-63] Miranda FR, Casali DM, Perini FA, Machado FA, Santos FR (2017). Taxonomic review of the genus *Cyclopes* Gray, 1821 (Xenarthra: Pilosa), with the revalidation and description of new species. Zoological Journal of the Linnean Society.

[ref-64] Mook CC (1942). A new fossil crocodilian from the Paleocene of New Mexico. American Museum Novitates.

[ref-65] Mook CC, Mook GE (1940). Some problems in crocodilian nomenclature. American Museum Novitates.

[ref-66] Moreno-Bernal JW, Head J, Jaramillo CA (2016). Fossil crocodilians from the high Guajira pennisula of Colombia: Neogene faunal change in the northernmost South America. Journal of Vertebrate Paleontology.

[ref-67] Müller J, Reisz RR (2005). Four well-constrained calibration points from the vertebrate fossil record for molecular clock estimates. BioEssays.

[ref-68] Muniz F, Bittencourt PS, Farias IP, Hrbek T, Campos Z (2015). New Records on Occurrence of *Paleosuchus* in the Branco River Basin, Roraima State, Brazil. Crocodile Specialist Group Newsletter.

[ref-69] Muniz FL, Campos Z, Hernández Rangel SM, Martínez JG, Souza BC, De Thoisy B, Botero-Arias R, Hrbek T, Farias IP (2018). Delimitation of evolutionary units in Cuvier’s dwarf caiman, *Paleosuchus palpebrosus* (Cuvier, 1807): insights from conservation of a broadly distributed species. Conservation Genetics.

[ref-70] Murphy JC, Jowers MJ, Lehtinen RM, Charles SP, Colli GR, Peres Jr AK, Hendry CR, Pyron RA (2016). Cryptic, sympatric diversity in tegu lizards of the *Tupinambis teguixin* group (Squamata, Sauria, Teiidae) and the description of three new species. PLOS ONE.

[ref-71] Nater A, Mattle-Greminger MP, Nurcahyo A, Nowak MG, De Manuel M, Desai T, Groves C, Pybus M, Sonay TB, Roos C, Lameira AR, Wich SA, Askew J, Davila-Ross M, Fredriksson G, De Valles G, Casals F, Prado-Martinez J, Goossens B, Verschoor EJ, Warrens KS, Singleton I, Marques DA, Pamungkas J, Perwitasari-Farajallah D, Rianti P, Tuuga A, Gut IG, Gut M, Orozco-terWengel 1, Van Schaik CP, Bertranpetit J, Anisimova M, Scally A, Marques-Bonet T, Meijaard E, Krutzen M (2017). Morphometric, behavioral, and genomic evidence for a new Orangutan species. Current Biology.

[ref-72] Oaks JR (2011). A time-calibrated species tree of Crocodylia reveals a recent radiation of the true crocodiles. Evolution.

[ref-73] Pääbo S, Higuchi RG, Wilson AC (1989). Ancient DNA and the polymerase chain reaction. Journal of Biological Chemistry.

[ref-74] Pfenninger M, Schwenk K (2007). Cryptic animal species are homogeneously distributed among taxa and biogeographical regions. BMC Evolutionary Biology.

[ref-75] Posada D (2008). jModelTest: phylogenetic model averaging. Molecular Biology and Evolution.

[ref-76] Poulakakis N, Edwards DL, Chiari Y, Garrick RC, Russello MA, Benavides E, Watkins-Colwell GJ, Glaberman S, Tapia W, Gibbs JP, Cayot LJ, Caccone A (2015). Description of a new galapagos giant tortoise species (*Chelonoidis*; Testudines: Testudinidae) from Cerro Fatal on Santa Cruz Island. PLOS ONE.

[ref-77] R Development Core Team (2011).

[ref-78] Rambaut A, Drummond AJ, Xie D, Baele G, Suchard MA (2018). Posterior summarization in bayesian phylogenetics using Tracer 1.7. Systematic Biology.

[ref-79] Reid NM, Carstens BC (2012). Phylogenetic estimation error can decrease the accuracy of species delimitation: a Bayesian implementation of the general mixed Yule-coalescent model. BMC Evolutionary Biology.

[ref-80] Rice WR (1989). Analyzing tables of statistical tests. Evolution.

[ref-81] Riff D, Romano PSR, Oliveira GR, Aguilera OA, Hoorn C, Wesselingh FP (2009). Neogene crocodile and turtle fauna in northern South America. Amazonia: landscape and species evolution.

[ref-82] Roca AL, Georgiadis NJ, Pecon-Slattery J, O’Brien SJ (2001). Genetic evidence for two species of elephant in Africa. Science.

[ref-83] Roos J, Aggarwal RK, Janke A (2007). Extended mitogenomic phylogenetic analyses yield new insight into crocodylian evolution and their survival of the Cretaceous-Tertiary boundary. Molecular Phylogenetics and Evolution.

[ref-84] Rull V (2011). Neotropical biodiversity: timing and potential drivers. Trends in Ecology & Evolution.

[ref-85] Salas-Gismondi R, Flynn JJ, Baby P, Tejada-Lara JV, Wesselingh FP, Antoine PO (2015). A Miocene hyperdiverse crocodylian community reveals peculiar trophic dynamics in proto-Amazonian mega-wetlands. Proceedings of the Royal Society B.

[ref-86] Salzburger W, Ewing GB, Von Haeseler A (2011). The performance of phylogenetic algorithms in estimating haplotype genealogies with migration. Molecular Ecology.

[ref-87] Schaefer CER, Dalrymple J (1996). Pedogenesis and relict properties of soils with columnar structure from Roraima, north Amazonia. Geoderma.

[ref-88] Schaefer CER, Vale Júnior JF, Barbosa RI, Ferreira EJG, Castellón EG (1997). Mudanças climáticas e evolução da paisagem em Roraima: uma resenha do Cretáceo ao recente. Homem, Ambiente e Ecologia no Estado de Roraima.

[ref-89] Scheyer TM, Aguilera OA, Delfino M, Fortier DC, Carlini AA, Sánchez R, Carrillo-Briceño JD, Quiroz L, Sánchez-Villagra MR (2013). Crocodylian diversity peak and extinction in the late Cenozoic of the northern Neotropics. Nature Communications.

[ref-90] Schliep KP (2011). phangorn: phylogenetic analysis in R. Bioinformatics.

[ref-91] Schmidt KP (1928). Notes on south american caimans. Field Museum of Natural History, Publication 252, Zoological Series.

[ref-92] Scholl JP, Wiens JJ (2016). Diversification rates and species richness across the Tree of Life. Proceedings of the Royal Society B: Biological Sciences.

[ref-93] Shirley MH, Carr AN, Nestler JH, Vliet KA, Brochu CA (2018). Systematic revision of the living African Slender-snouted Crocodiles (*Mecistops* Gray, 1844). Zootaxa.

[ref-94] Shirley MH, Vliet KA, Carr AN, Austin JD (2014). Rigorous approaches to species delimitation have significant implications for African crocodilian systematics and conservation. Proceedings of the Royal Society B: Biological Sciences.

[ref-95] Simula R, Schulte R, Summers K (2003). Molecular systematics and phylogeography of Amazonian poison frogs of the genus *Dendrobates*. Molecular Phylogenetics and Evolution.

[ref-96] Solórzano A, Rincón AD, Cidade GM, Núñez Flores M, Sánchez L (2018). Lower Miocene alligatoroids (Crocodylia) from the Castillo Formation, northwest of Venezuela. Palaeobiodiversity and Palaeoenvironments.

[ref-97] Souza-Mazurek RR (2001). Habitat, *Paleosuchus trigonatus*. Herpetological Review.

[ref-98] Tajima F (1989). Statistical method for testing the neutral mutation hypothesis by DNA polymorphism. Genetics.

[ref-99] Thompson JD, Higgins DG, Gibson TJ (1996). CLUSTAL W: improving the sensitivity of progressive multiple sequence alignment through sequence weighting, position specific gap penalties and weight matrix choice. Nucleic Acids Research.

[ref-100] Turchetto-Zolet AC, Pinheiro F, Salgueiro F, Palma-Silva C (2012). Phylogeographical patters shed light on evolutionary process in South America. Molecular Ecology.

[ref-101] Vasconcelos W, Campos Z (2007). Geographic variation between Pantanal caiman (*Caiman crocodilus yacare*) and Amazonian caiman (*Caiman crocodilus crocodilus*): first phase. Crocodile Specialist Group Newsletter.

[ref-102] Vasconcelos WR, Hrbek T, Da Silveira R, Thoisy B, Marioni B, Farias IP (2006). Population genetic analysis of *Caiman crocodilus* (Linnaeus, 1758) from South America. Genetics and Molecular Biology.

[ref-103] Vasconcelos WR, Hrbek T, Da Silveira R, Thoisy B, Ruffeil LAAS, Farias IP (2008). Phylogeographic and conservation genetic analysis of the black caiman (*Melanosuchus niger*). Journal of Experimental Zoology.

[ref-104] Venables WN, Ripley BD (2002). Modern applied statistics with S.

[ref-105] Venegas-Anaya M, Crawford AJ, Escobedo-Galván AH, Sanjur OI, Densmore IIILD, Bermingham E (2008). Mitochondrial DNA phylogeography of *Caiman crocodilus* in Mesoamerica and South America. Journal of Experimental Zoology.

[ref-106] Weir BS, Cockerham CC (1984). Estimating F-statistics for the analysis of population structure. Evolution.

[ref-107] Williamson TE (1996). ?*Brachychampsa Sealeyi*, Sp Nov. (Crocodylia, Alligatoroidea) From the Upper Cretaceous (Lower Campanian) Menefee Formation, Northwestern New Mexico. Journal of Vertebrate Paleontology.

[ref-108] Willis SC (2017). One species or four? Yes!...and, no. Or, arbitrary assignment of lineages to species obscures the diversification processes of Neotropical fishes. PLOS ONE.

[ref-109] Yu G, Smith DK, Zhu H, Guan Y, Lam TT-Y (2017). ggtree: an r package for visualization and annotation of phylogenetic trees with their covariates and other associated data. Methods in Ecology and Evolution.

