# Supplementary material for: Evidence of cryptic lineages within a small South American crocodilian: the Schneider’s dwarf caiman Paleosuchus trigonatus (Alligatoridae: Caimaninae)
Source: PeerJ. 2019 Mar 22;7:e6580. doi: 10.7717/peerj.6580 (PMC6433001; doi:10.7717/peerj.6580)
Supplement: Supplementary file 1 [file peerj-07-6580-s001.odt]

Supplemental Table 1. Sampling localities and number of samples (N) of Paleosuchus trigonatus utilized in this study.

| Locality | Coordinates | N | River drainage |
| --- | --- | --- | --- |
| 1. Amajari | 3°50' N 61°26' W | 2 | Upper Branco |
| 2. Maracá Ecological Station | 3°21' N 61°26' W | 25 | Upper Branco |
| 3. Mucajaí | 2°28' N 60°55' W | 2 | Upper Branco |
| 4. Cantá | 2°27' N 60°45' W | 4 | Upper Branco |
| 5. Viruá National Park | 1°29' N 60°58' W | 1 | Middle Branco |
| 6. Itã river | 1°28' N 60°42' W | 1 | Middle Branco |
| 7. French Guiana | 2°55' N 53°38' W | 6 | Tampok |
| 8. Calçoene | 2°26' N 51°24' W | 1 | Calçoene |
| 9. Porto Grande | 0°35'N 52°19' W | 3 | Amapari |
| 10. Jundiá village | 0°5' S 60°41' W | 5 | Lower Negro |
| 11. Waimiri-Atroari Indigenous Land | 0°48' S 60°32' W | 6 | Lower Negro |
| 12. Canoas stream | 1°49' S 60°11' W | 5 | Lower Negro |
| 13. Presidente Figueiredo | 2°7' S 59°56' W | 2 | Urubu |
| 14. Balbina District | 1°56' S 59°25' W | 2 | Uatuma |
| 15. UFAM Experimental Farm | 2°39' S 60°3' W | 2 | Lower Negro |
| 16. Manaus | 3°6' S 59°57' W | 23 | Lower Negro |
| 17. Mãe Cué Community | 1°27' S 56°44' W | 1 | Trombetas |
| 18. Vitória do Xingu | 3°4' S 51°46' W | 6 | Xingu |
| 19. Altamira | 3°12' S 52°12' W | 15 | Xingu |
| 20. Iriri river | 3°51' S 52°51' W | 1 | Xingu |
| 21. São Pedro da Água Branca | 5°6' S 48°20' W | 1 | Tocantins |
| 22. Novo Progresso | 7°3' S 55°27' W | 1 | Jamanxim |
| 23. Cachoeira da Serra | 8°36' S 55°1' W | 1 | Iriri |
| 24. Campos Novos dos Parecis | 12°47' S 57°42' W | 2 | Juruena |
| 25. São José do Rio Claro | 13°31' S 56°37' W | 1 | Juruena |
| 26. Japurá river | 1°50' S 69°2' W | 3 | Japurá |
| 27. Pacaya-Samiria National Reserve | 5°13' S 74°34' W | 2 | Ucayali |
| 28. Pucallpa | 8°23' S 74°32' W | 1 | Ucayali |
| 29. Puerto Maldonado | 12°35' S 69°10' W | 2 | Tambopata |
| 30. Beruri | 4°17' S 61°40' W | 23 | Lower Purus |
| 31. Canutama | 6°14' S 64°27' W | 1 | Middle Purus |
| 32. Manicoré | 5°52' S 61°15' W | 3 | Aripuanã |
| 33. Aripuanã river | 7°14' S 60°39' W | 8 | Aripuanã |
| 34. Humaitá | 7°33' S 63°16' W | 10 | Madeira |
| 35. Porto Velho | 8°48' S 63°57' W | 6 | Madeira |
| 36. Madeira between rapids | 9°7' S 64°32' W | 11 | Madeira |
| 37. Jaci-Paraná river | 9°17' S 64°23' W | 24 | Madeira |
| 38. Fortaleza do Abunã | 9°45' S 65°30' W | 9 | Upper Madeira |
| 39. Nova Mamoré | 10°42' S 65°24' W | 4 | Upper Madeira |
| 40. Guajará-mirim | 10°49' S 65°18' W | 2 | Upper Madeira |
| 41. São Miguel do Guaporé | 11°43' S 62°48' W | 1 | Upper Madeira |
| 42. Cacoal | 11°29' S 61°26' W | 1 | Upper Madeira |
| Total |  | 230 |  |
