# Supplementary material for: Evidence of cryptic lineages within a small South American crocodilian: the Schneider’s dwarf caiman Paleosuchus trigonatus (Alligatoridae: Caimaninae)
Source: PeerJ. 2019 Mar 22;7:e6580. doi: 10.7717/peerj.6580 (PMC6433001; doi:10.7717/peerj.6580)
Supplement: Supplementary file 2 [file peerj-07-6580-s002.odt]

Supplemental Table 2. Haplotypes (H), polymorphic sites, nucleotide position, and number of individuals per haplotype (N) sampled in this study. 

| H | Nucleotide Position | N |
| --- | --- | --- |
|  |  |  |  |  |  |  |  |  |  |  |  |  |  |  |  |  |  |  |  |  |  |  |  |  |  |  |  |  |  |  |  |  |  |  |  |  |  |  |  |  |  |  |  |  |  |  |  |  |  |  |  |  |  |  |  | 1 | 1 |  |
|  |  |  |  |  |  |  |  |  |  | 1 | 1 | 2 | 2 | 2 | 2 | 3 | 3 | 3 | 3 | 3 | 4 | 4 | 4 | 4 | 5 | 5 | 5 | 5 | 5 | 6 | 6 | 6 | 6 | 6 | 6 | 7 | 7 | 7 | 7 | 7 | 7 | 8 | 8 | 8 | 8 | 8 | 8 | 8 | 9 | 9 | 9 | 9 | 9 | 9 | 9 | 0 | 0 |  |
|  | 1 | 1 | 3 | 3 | 3 | 3 | 4 | 6 | 8 | 2 | 3 | 3 | 4 | 9 | 9 | 1 | 5 | 6 | 7 | 8 | 4 | 6 | 7 | 9 | 1 | 2 | 2 | 2 | 7 | 1 | 1 | 4 | 4 | 6 | 7 | 1 | 2 | 6 | 6 | 7 | 9 | 0 | 2 | 3 | 6 | 7 | 8 | 9 | 0 | 0 | 3 | 4 | 6 | 7 | 9 | 0 | 0 |  |
|  | 3 | 6 | 0 | 3 | 4 | 5 | 0 | 0 | 1 | 9 | 0 | 4 | 3 | 4 | 7 | 5 | 7 | 7 | 2 | 4 | 4 | 5 | 1 | 8 | 0 | 0 | 3 | 9 | 4 | 6 | 9 | 0 | 1 | 9 | 7 | 7 | 6 | 3 | 8 | 6 | 3 | 5 | 2 | 8 | 4 | 7 | 2 | 7 | 1 | 6 | 7 | 8 | 9 | 0 | 9 | 8 | 9 |  |
| H1 | T | A | A | A | G | C | C | G | A | G | G | T | C | G | A | G | C | A | G | G | A | G | T | A | C | C | A | G | A | A | C | A | G | C | C | T | C | C | T | C | C | A | G | G | C | C | C | G | A | A | A | C | C | A | A | G | G | 8 |
| H2 | . | . | . | . | . | . | . | . | . | . | . | . | . | . | . | . | . | . | . | . | . | . | . | . | . | . | . | . | . | . | . | . | . | . | . | . | . | T | . | . | . | . | . | . | . | . | . | . | . | . | . | . | . | . | . | . | . | 1 |
| H3 | C | . | . | . | . | . | . | . | . | . | . | . | . | A | . | . | . | G | . | . | . | . | . | . | . | . | . | . | . | . | . | . | . | . | . | . | . | . | . | . | . | . | . | . | . | . | . | . | . | . | . | . | . | . | . | . | . | 1 |
| H4 | C | . | . | . | . | . | . | . | . | . | . | . | . | . | . | . | . | . | . | . | . | . | . | . | T | . | . | . | . | . | . | . | . | . | . | . | . | . | C | . | . | . | . | . | . | . | . | . | . | . | . | . | . | . | G | . | . | 32 |
| H5 | C | . | . | . | . | . | . | . | . | . | . | . | . | . | . | . | . | . | . | . | . | . | . | C | T | . | . | . | . | . | . | . | . | . | . | . | . | . | C | . | . | . | . | . | . | . | . | . | . | . | . | . | . | . | G | . | . | 1 |
| H6 | C | . | . | . | . | . | . | . | . | . | . | . | . | . | . | . | . | . | . | . | . | . | . | . | T | . | . | . | . | . | . | T | C | . | . | . | . | . | C | . | . | . | . | . | . | . | . | . | . | . | . | . | . | . | G | . | . | 1 |
| H7 | C | . | T | G | . | T | . | A | G | A | A | A | A | A | . | A | . | . | A | . | C | . | C | . | . | . | G | A | G | G | T | . | . | . | . | C | . | . | C | T | T | G | A | . | T | T | . | A | . | G | . | . | . | G | . | A | A | 41 |
| H8 | C | . | . | G | . | T | . | A | G | A | A | A | A | A | . | A | . | . | A | . | C | . | C | . | . | . | G | A | G | G | T | . | . | . | . | C | . | . | C | T | T | G | A | . | . | T | . | A | . | G | G | . | . | G | G | A | A | 68 |
| H9 | C | . | . | G | . | T | . | A | G | A | A | A | A | A | . | A | . | . | A | . | C | . | C | . | . | . | G | A | G | G | T | . | . | . | . | C | . | . | C | T | T | G | A | . | . | . | . | A | . | G | G | . | . | G | G | A | A | 2 |
| H10 | C | . | . | . | . | T | . | A | G | A | A | A | A | A | . | A | . | . | A | . | C | . | C | . | . | . | G | A | G | G | T | . | . | . | . | C | . | . | C | T | T | G | A | . | . | T | . | A | . | G | G | . | . | G | G | A | A | 7 |
| H11 | C | . | . | G | . | T | . | A | G | A | A | A | A | A | . | . | . | . | A | . | C | . | C | . | . | . | G | A | G | G | T | . | . | . | . | C | . | . | C | T | T | G | A | . | . | T | . | A | . | G | G | . | . | G | G | A | A | 2 |
| H12 | C | . | . | G | . | T | . | A | G | A | A | A | A | A | . | A | T | . | A | . | C | . | C | . | . | . | G | A | G | G | T | . | . | . | . | C | . | . | C | T | T | G | A | . | . | T | . | A | . | G | G | . | . | G | G | A | A | 1 |
| H13 | C | . | G | G | . | T | . | A | G | A | A | A | A | A | . | A | . | . | A | . | C | . | C | . | . | . | G | A | G | G | T | . | . | . | . | C | . | . | C | T | T | G | A | . | T | T | . | A | . | G | . | T | . | G | . | A | A | 1 |
| H14 | C | . | T | G | . | T | . | A | G | A | A | A | A | A | . | A | . | . | A | . | C | . | C | . | . | . | G | A | G | G | T | . | . | . | . | C | . | . | C | T | T | G | A | . | T | T | . | A | G | G | . | . | . | G | . | A | A | 1 |
| H15 | C | . | G | G | . | T | . | A | G | A | A | A | A | A | G | A | . | . | A | . | C | . | C | . | . | . | G | A | G | G | T | . | . | . | . | C | . | . | C | T | T | G | A | . | T | T | . | A | . | G | . | . | . | G | . | A | A | 7 |
| H16 | C | . | G | G | . | T | . | A | G | A | A | A | A | A | . | A | . | . | A | . | C | . | C | . | . | . | G | A | G | G | T | . | . | . | . | C | . | . | C | T | T | G | A | . | T | T | . | A | . | G | . | . | . | G | . | A | A | 3 |
| H17 | C | . | G | G | . | T | T | A | G | A | A | A | A | A | . | A | . | . | A | . | C | . | C | . | . | . | G | A | G | G | T | . | . | . | . | C | . | . | C | T | T | G | A | . | T | T | . | A | . | G | . | . | . | G | . | A | A | 9 |
| H18 | C | . | . | G | . | T | . | A | G | A | A | A | A | A | . | A | . | . | A | . | C | A | C | . | . | . | G | A | G | G | T | . | . | . | . | C | . | . | C | T | T | G | A | . | . | T | . | A | . | G | G | . | T | G | G | A | A | 1 |
| H19 | C | . | . | G | . | T | . | A | G | A | A | A | A | A | . | A | . | . | A | . | C | A | C | . | . | . | G | A | G | G | T | . | . | . | . | C | . | . | C | T | T | G | A | . | . | T | . | A | . | G | G | . | . | G | G | A | A | 1 |
| H20 | C | G | . | G | . | T | . | A | G | A | A | A | A | A | . | A | . | . | A | . | C | . | C | . | . | . | G | A | G | G | T | . | . | . | . | C | . | . | C | T | T | G | A | . | . | T | T | A | . | G | G | . | . | G | G | A | A | 1 |
| H21 | C | . | . | G | . | T | . | A | G | A | A | A | A | A | . | A | . | . | A | . | C | . | C | . | . | . | G | A | G | G | T | . | . | . | . | C | . | . | C | T | T | G | A | . | . | T | T | A | . | G | G | . | . | G | G | A | A | 3 |
| H22 | C | . | T | G | . | T | . | A | G | A | A | A | A | A | . | A | . | . | A | . | C | . | C | . | . | . | G | A | G | G | T | . | . | . | . | C | . | . | C | T | T | G | A | . | T | T | . | A | . | G | . | . | . | . | . | A | A | 1 |
| H23 | C | . | T | G | A | T | . | A | G | A | A | A | A | A | . | A | . | . | A | . | C | . | C | . | . | . | G | A | G | G | T | . | . | . | . | C | . | . | C | T | T | G | A | . | T | T | . | A | . | G | . | . | . | G | . | A | A | 11 |
| H24 | C | . | . | G | . | T | . | A | G | A | A | A | A | A | . | A | . | . | A | . | C | . | C | . | . | . | G | A | G | G | T | . | . | . | T | C | . | . | C | T | T | G | A | . | . | T | . | A | . | G | G | . | . | G | G | A | A | 5 |
| H25 | C | . | T | G | . | T | . | A | G | A | A | A | A | A | . | . | . | . | A | . | C | . | C | . | . | . | G | A | G | G | T | . | . | . | . | C | . | . | C | T | T | G | A | . | T | T | . | A | . | G | . | . | . | G | . | A | A | 2 |
| H26 | C | . | T | G | . | T | . | A | G | A | A | A | A | A | . | A | . | . | A | . | C | . | C | . | . | . | G | A | G | G | T | . | . | . | . | C | . | . | C | T | T | G | A | A | T | T | . | A | . | G | . | . | . | . | . | A | A | 3 |
| H27 | C | . | T | G | . | T | . | A | G | A | A | A | A | A | . | A | . | . | A | . | C | . | C | . | . | . | G | A | G | G | T | . | . | . | . | C | . | . | C | T | T | G | A | A | T | T | . | A | . | G | . | . | . | G | . | A | A | 1 |
| H28 | C | . | . | G | . | T | . | A | G | A | A | A | A | A | . | A | . | . | A | . | C | . | C | . | . | . | G | A | G | G | T | . | . | . | . | C | . | . | C | T | T | G | A | . | . | T | . | A | . | G | G | . | . | . | G | A | A | 1 |
| H29 | C | . | . | G | . | T | . | A | G | A | A | A | A | A | . | A | . | . | A | . | C | . | C | . | . | T | G | A | G | G | T | . | . | . | . | C | T | . | C | T | T | G | A | . | T | T | . | A | . | G | . | . | . | G | . | A | A | 3 |
| H30 | C | . | T | G | A | T | T | A | G | A | A | A | A | A | . | A | . | . | A | . | C | . | C | . | . | . | G | A | G | G | T | . | . | . | . | C | . | . | C | T | T | G | A | . | T | T | . | A | . | G | . | . | . | G | . | A | A | 1 |
| H31 | C | . | G | G | . | T | . | A | G | A | A | A | A | A | . | A | . | . | A | . | C | . | C | . | . | . | G | A | G | G | T | . | . | . | . | C | . | . | C | T | T | G | A | . | T | T | . | A | . | G | G | . | . | G | . | A | A | 1 |
| H32 | C | . | T | G | . | T | . | A | G | A | A | A | A | A | . | A | . | . | A | . | C | . | C | . | . | . | G | A | G | G | T | . | . | . | . | C | . | . | C | T | T | G | A | . | T | T | . | A | . | G | . | . | . | G | G | A | A | 1 |
| H33 | C | . | G | G | . | T | . | A | G | A | A | A | A | A | . | A | . | . | A | A | C | . | C | . | . | . | G | A | G | G | T | . | . | . | . | C | . | . | C | T | T | G | A | . | T | T | . | A | . | G | . | . | . | G | . | A | A | 1 |
| H34 | . | . | . | G | . | T | . | A | G | A | A | A | A | A | . | A | . | . | A | . | C | . | C | . | . | . | G | A | G | G | T | . | . | . | . | C | . | . | C | T | T | G | A | . | . | T | . | A | . | G | G | . | . | G | G | A | A | 1 |
| H35 | . | G | . | G | . | T | . | A | G | A | A | A | A | A | . | A | . | . | A | . | C | . | C | . | . | . | G | A | G | G | T | . | . | T | . | C | . | . | C | T | T | G | A | . | . | T | . | A | . | G | G | . | . | G | G | A | A | 5 |
| H36 | . | G | . | G | . | T | . | A | G | A | A | A | A | A | . | A | . | . | A | . | C | . | C | . | . | . | G | A | G | G | T | . | . | . | . | C | . | . | C | T | T | G | A | . | . | T | . | A | . | G | G | . | . | G | G | A | A | 1 |
